# Clinical value of a plasma Epstein–Barr virus DNA assay in the diagnosis of recurrent or metastatic nasopharyngeal carcinoma: a meta-analysis

**DOI:** 10.1042/BSR20190691

**Published:** 2019-09-20

**Authors:** Haiqin Peng, Zhanzhan Li, Yujiao Long, Jiahui Li, Zhiyuan Liu, Rongrong Zhou

**Affiliations:** Department of Oncology, Xiangya Hospital, Central South University, Changsha 410008, China

**Keywords:** Epstein-Barr Virus DNA, Meta-Analysis, metastatic, nasopharyngeal carcinoma, recurrent

## Abstract

Background: To evaluate the diagnostic value of Epstein–Barr virus (EBV) DNA in nasopharyngeal carcinoma (NPC) patients with locoregional or distant recurrence.

Methods: Articles related to the diagnosis of recurrent or metastatic NPC by the detection of EBV DNA in plasma or serum were retrieved from different databases. Sensitivity, specificity, summary receiver operating characteristic (SROC) curves, and likelihood ratios were pooled to assess the diagnostic value of individual diagnostic tests.

Results: This meta-analysis pooled 25 eligible studies including 2496 patients with NPC. The sensitivity, specificity, positive likelihood ratio (+LR), and negative likelihood ratio (−LR) of EBV DNA in the diagnosis of NPC were 0.858 (95% confidence interval (CI): 0.801–0.901), 0.890 (95% CI: 0.866–0.909), 7.782 (95% CI: 6.423–9.429) and 0.159 (95% CI: 0.112–0.226), respectively. The diagnostic odds ratio (DOR) was 48.865 (95% CI: 31.903–74.845). The SROC for EBV DNA detection was 0.93 (95% CI: 0.90–0.95).

Conclusion: The detection of EBV DNA for the diagnosis of recurrent or metastatic NPC has good sensitivity and specificity and might be helpful in monitoring recurrent or metastatic NPC.

## Introduction

Nasopharyngeal carcinoma (NPC) is a type of cancer with a particularly high incidence in Southern China and Southeast Asian countries, affecting 10–50 per 100000 people per year [1–4]. The standard therapy for NPC is radiotherapy and concurrent chemoradiotherapy (CCRT) depending on the stage of disease during presentation [5]. Despite significant improvements in survival and local control due to advances in radiotherapy and combined modality treatments, local recurrence and distant metastasis remain difficult to avoid in patients with advanced NPC [6]. It was reported that the rate of local recurrence and distant metastasis after 5 years of the initial treatment for NPC is 8.2–22.0% [7]. Currently, the main diagnostic method for the recurrence or metastasis of NPC patients is clinical imaging examination combined with endoscopic biopsy. However, a series of abnormal changes, including local edema, tissue disorder, fibrosis, mucositis, and scar formation, always occur in the post-treatment of NPC patients, which significantly interferes with the accuracy of an imaging examination [8–10]. In addition, with computed tomography (CT) and magnetic resonance imaging (MRI), it is difficult to detect distant metastases early and specifically when the diameter of the lesion is less than 5  mm [11], which will delay the discovery of the tumor. Meanwhile, the high cost of PET/CT examinations and the general application during follow-up are inconsistent with the economic development level of China. Pathological examination is the gold standard for the diagnosis of NPC recurrence and metastasis. Generally, it is difficult to obtain pathological sections of recurrent or metastatic lesions, especially those that occur under the mucosa or deep in the nasopharynx. More importantly, invasive procedures used to obtain pathological diagnoses are an important poor prognostic factor for the disease [12]. Therefore, seeking a more concise and effective diagnostic method would be of great importance for NPC patients.

NPC is strongly associated with Epstein–Barr virus (EBV). Plasma EBV DNA level has been used as a tumor marker for NPC and is widely used in clinical screening and diagnosis of NPC [13]. However, the value of plasma-free EBV DNA in the diagnosis of recurrence and metastasis of NPC is not clear currently. Different studies have found that there is a large difference in the critical value of free EBV DNA expression after treatment [14–17]. These differences may be due to experimental methods used by different investigators and geographical differences in NPC itself.

Due to insufficient research and inconsistent reports, there is no uniform and accurate conclusion on whether plasma EBV DNA can effectively detect the recurrence and metastasis of NPC. Therefore, we performed this meta-analysis to better assess the diagnostic value of plasma EBV DNA in recurrent or metastatic NPC patients.

## Methods

We conducted this meta-analysis on the basis of the Preferred Reporting Items for Systematic Reviews and Meta-Analyses (PRISMA) statement. All analyses were conducted based on previously published studies; thus, no ethical approval or patient consent were required.

### Search strategy

Two reviewers (Haiqin Peng and Zhanzhan Li) independently completed a search. There was no restriction on the language of the studies. The search strategy combined the following key words: (‘Epstein–Barr Virus’ [All Fields]) OR (‘EBV’ [All Fields]) OR (‘DNA’ [All Fields]) OR (‘EBV-DNA’ [All Fields]) OR (‘EBV DNA’ [All Fields]) OR (‘Epstein–Barr Virus DNA’ [All Fields]) AND (‘nasopharyngeal carcinoma’ [All Fields]) OR (‘nasopharyngeal cancer’ [All Fields]) OR (‘carcinoma of nasopharynx’ [All Fields]) OR (‘NPC’ [All Fields]) AND (‘sensitivity’ [All Fields]) OR (‘specificity’ [All Fields]) OR (‘false-negative’ [All Fields]) OR (‘false-positive’ [All Fields]) OR (‘diagnosis’ [All Fields]) OR (‘detection’ [All Fields]) OR (‘accuracy’ [All Fields]) AND (‘plasma’ [All Fields]) OR (‘serum’ [All Fields]) AND (‘relapse’ [All Fields]) OR (‘recurrence’ [All Fields]) OR (‘metastasis’ [All Fields]). We used this search strategy to search PubMed (https://www.ncbi.nlm.nih.gov/pubmed), Web of Science (https://www.webofknowledge.com), EMBASE (https://www.embase.com), the Chinese Biomedical Database (http://www.sinomed.ac.cn/zh/), and the China National Knowledge Infrastructure (http://www.cnki.net/) website for articles published from January 1998 to July 2018. References cited in the retrieved studies were reviewed for more eligible studies.

### Inclusion/exclusion criteria

Studies were considered eligible only when they met all of the following inclusion criteria: (1) the purpose of study was to evaluate the clinical value of EBV DNA in the diagnosis of NPC recurrence or metastasis; (2) identification of NPC was confirmed by histology or pathology; (3) the study clearly identified negative controls; and (4) the article provided data that can calculate true positive value (TP), false positive value (FP), true negative value (TN), false negative value (FN), directly or indirectly. If the data were repeatedly published, the most detailed data or the most recently published article were selected. The exclusion criteria were as follows: (1) studies that were published as review articles or letters; (2) the article lacking important information to calculate TP, FP, TN, and FN directly or indirectly; and (3) studies not clearly identifying negative controls.

### Data extraction

Two investigators reviewed the titles and abstracts of all records searched above to extract literature information that met the inclusion criteria. General information included study publication date and country, number of subjects, sample source, and study design. Any disagreements were discussed until a final form was agreed upon. For records that could not be evaluated by title and abstract, the full text was retrieved for detailed evaluation according to the inclusion and exclusion criteria. The data extracted from each study included basic characteristics of the studies and outcomes. Basic characteristics of the studies included the first author, year of the publication, country of origin, and sample size. Outcomes included the TP, FP, TN, and FN results calculated from each study.

### Quality assessment

The methodological quality of the selected studies was evaluated independently by two reviewers (Haiqin Peng and Zhanzhan Li) using the quality assessment of diagnostic accuracy studies (QUADAS) checklist [14–18]. This checklist includes 14 items: a representative spectrum (item 1), a clear selection criteria (item 2), an acceptable reference standard (item 3), an acceptable delay between tests (item 4), partial verification (item 5), the same reference test regardless of the index test result (item 6), incorporation bias (item 7), the execution of the index test in detail (item 8), the reference standard in detail (item 9), the index test results were blinded to the reference test results (item 10), the reference standard was blinded to the index test results (item 11), the availability of clinical data that would be available in clinical practice when using the index test (item 12), reporting of uninterpretable results (item 13), and an explanation of withdrawals from the study (item 14). The 14 items were assessed in all included articles, each of which was assessed as ‘yes’, ‘no’, or ‘unclear’. Disagreements were resolved by a third reviewer (Rongrong Zhou).

### Statistical analysis

Statistical analysis was conducted by using Review Manager 5.3.5 (Cochrane Collaboration, Oxford, U.K.) and STATA 12.0 software (Stata Corp, College Station, TX). The accuracy indexes of EBV DNA were pooled by meta-analysis, including sensitivity, specificity, positive likelihood ratio (LR+) and negative likelihood ratio (LR–), diagnostic odds ratio (DOR), and their 95% confidence interval (CI). A summary receiver operating characteristic (SROC) curve was used to evaluate the global summary of test performance, and the area under the SROC curve presents the overall performance of the detection method. An area under the SROC curve of 1.0 (100%) indicates perfect discriminatory ability. Heterogeneity across studies was assessed using Cochran’s Q test and *I^2^* statistics [19]. Heterogeneity was considered statistically significant when *P*<0.05 or *I^2^* > 50%. A fixed-effect model was used when there was no evidence of significant heterogeneity. Otherwise, a random-effect model was applied. Subgroup analysis was conducted to explore the possible sources of heterogeneity. All *P*-values were two-sided, and *P*<0.05 was considered statistically significant.

### Subgroup analysis

Subgroup analyses were performed based on the total number of subjects (sample <30 vs ≥30), study design (case–control vs cohort study), and test specimens (serum vs plasma) to investigate differences in sensitivity and specificity between subgroups.

## Results

### Literature selection

**Figure 1:**
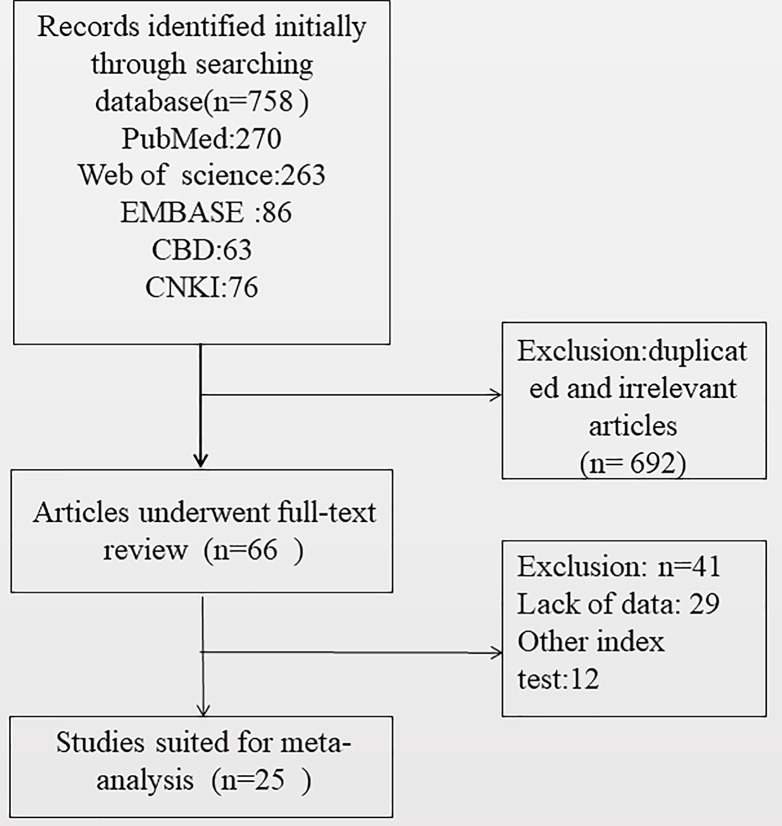
Flow chart of the selection process for eligible studies

The results of the literature research are presented in Figure 1. A total of 758 records were selected by searching the databases. After reviewing the titles and abstracts of studies, we excluded 733 studies. Finally, 25 studies [20–44] with 2496 samples were included in the final analysis. Among them, 20 papers were from China, which is consistent with the high incidence of NPC in China. The sample size of the studies ranged from 20 to 385. In addition, the gender ratio of the patients was reported in 13 studies, with a total of 1369 patients, of which 946 were males and 423 were females.

### Characteristics of studies

**Figure 2:**
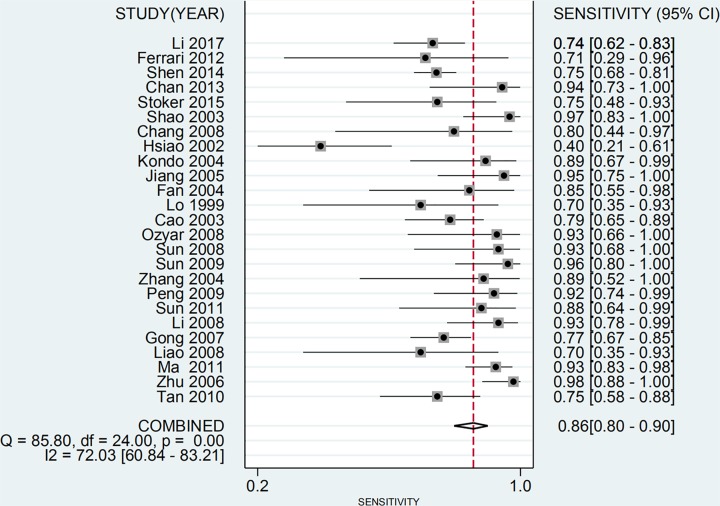
Forest plots of pooled sensitivity for EBV DNA assay in the recurrence or/and metastasis of NPC

**Figure 3:**
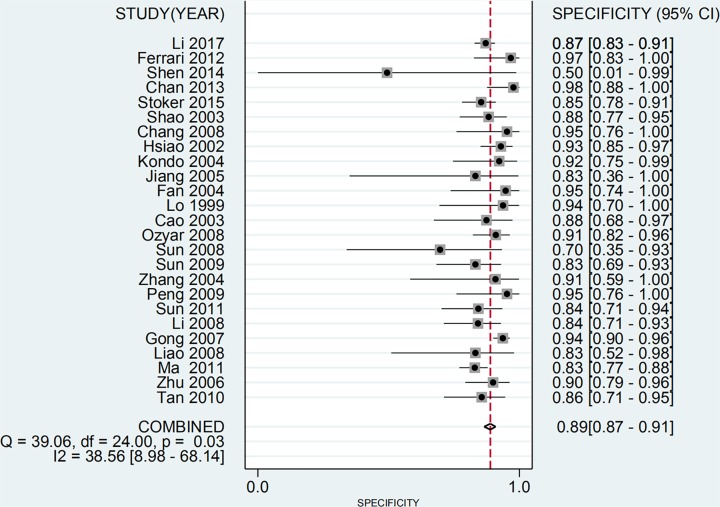
Forest plots of pooled specificity for EBV DNA assay in the recurrence or/and metastasis of NPC

**Figure 4:**
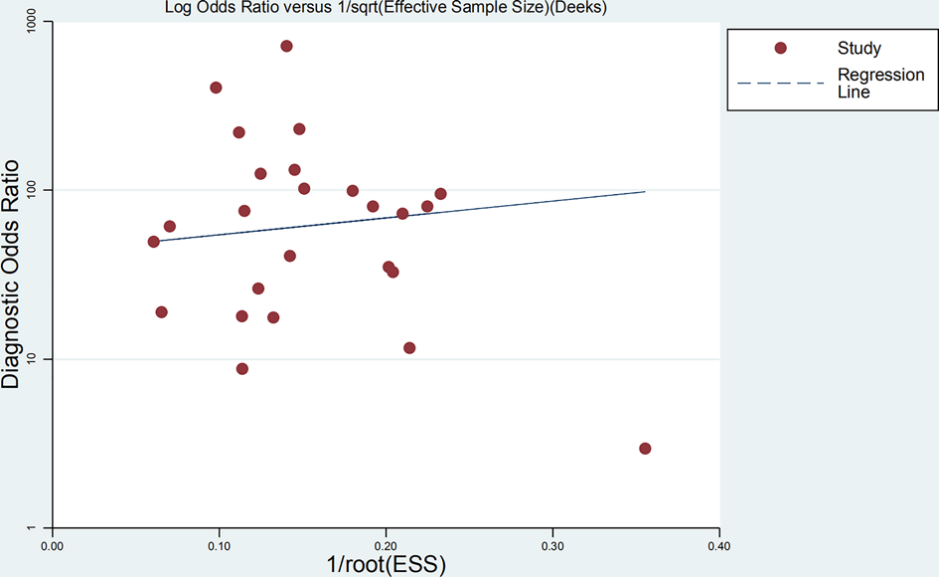
Forest plots of pooled DOR for EBV DNA assay in the recurrence or/and metastasis of NPC

**Figure 5:**
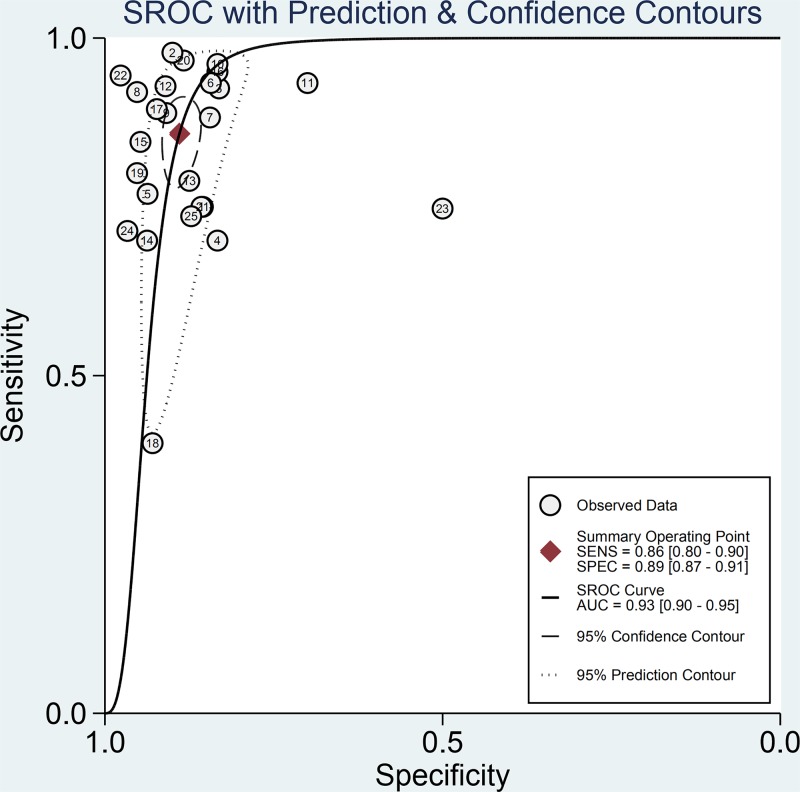
Summary ROC curve of all included articles with 95% CIs for pooled sensitivity and pooled specificity and the 95% prediction interval

**Figure 6:**
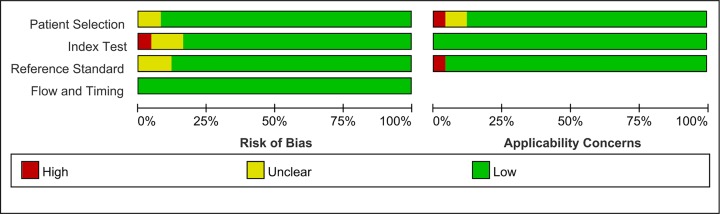
Risk of bias and applicability concerns graph

**Figure 7:**
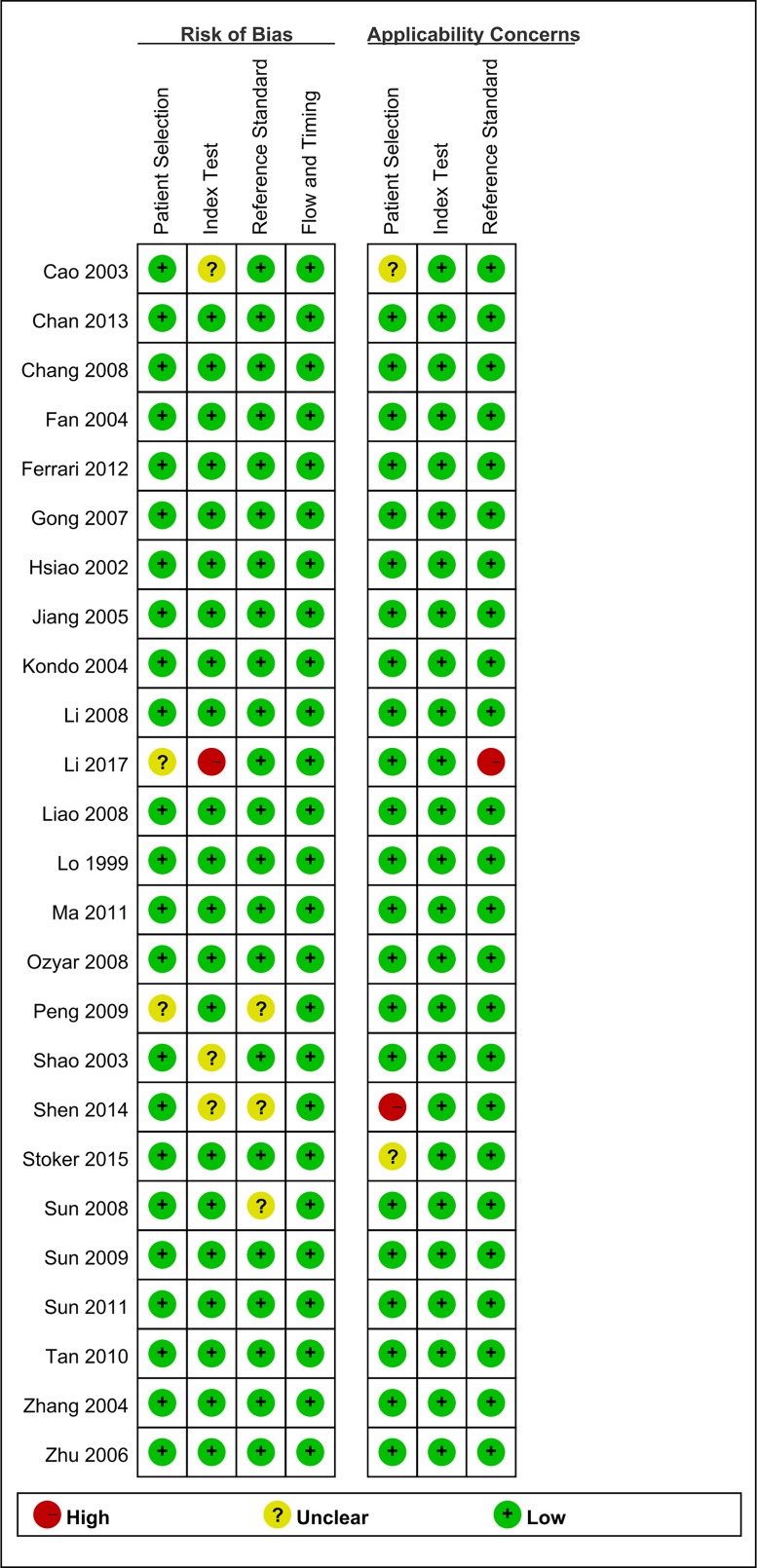
Risk of bias and applicability concerns summary

The main characteristics of the studies included in the meta-analysis are shown in Table 1. Among them, 22 study samples were from patients’ plasma and 3 study samples were from patients’ serum. In addition, 9 studies were case–control and 16 were cohort studies. The value of the plasma EBV DNA in diagnosing recurrent or metastatic NPC and the basic characteristics (TP, FP, TN, and FN values for serum EBV DNA) are shown in Table 2. We analyzed the pooled sensitivity, specificity, DOR, positive likelihood (+LR) and likelihood negative (−LR) of EBV DNA. Summary of meta-analysis results are shown in Table 3. The pooled results for sensitivity and specificity were 0.858 (95% CI: 0.801–0.901, Figure 2) and 0.890 (95% CI: 0.866–0.909, Figure 3), respectively. The highest sensitivity was 0.98 (95% CI: 0.88–1.0), which came from Zhu et al.’s study [21]. The lowest sensitivity was 0.40 (95% CI: 0.21–0.61), which came from Hsiao et al.’s study [37]. The highest specificity was 0.98 (95% CI: 0.88–1.0), which came from Chan et al.’s study [41]. The lowest specificity was 0.50 (95% CI: 0.01–0.99), which came from Shen et al.’s study [42]. The value of the DOR was 48.865 (95% CI: 31.903–74.845, Figure 4), which reflects the extent of the association between the results of diagnostic tests and diseases. Fagan diagram (Supplementary Figure S1.) also indicated the plasma EBV DNA had a high diagnostic ability in detecting recurrence or metastasis NPC. In addition, we also calculated LR+ and LR−, which are considered to be more clinically meaningful than sensitivity or specificity, to measure the diagnostic performance of the plasma EBV DNA in NPC with recurrence or metastasis. The pooled results of LR+ and LR− were 7.782 (95% CI: 6.423–9.429) and 0.159 (95% CI: 0.112 –0.226), respectively. The largest area of diagnosis under the summary receiver operator curve (AUC) for NPC by overall EBV DNA detection was 0.93 (95% CI: 0.90–0.95, Figure 5), indicating a relatively high accuracy. According to the QUADS scale, green stands for low risk, red stands for high risk, and yellow stands for risk unclear. Risk of bias and applicability concerns graph is presented in Figure 6. Risk of bias and applicability concerns summary is presented in Figure 7.

**Table 1:** Characteristics of studies included in the meta-analysis

Study ID	Year	Region	Number	Sample source	Sampling consecutive	Data collection retrospective	Study design
Tan et al. [20]	2010	China	78	Plasma	Yes	No	Cohort study
Zhu et al. [21]	2006	China	106	Plasma	Yes	No	Cohort study
Ma et al. [22]	2011	China	274	Plasma	Yes	No	Cohort study
Liao et al. [23]	2008	China	22	Plasma	Yes	No	Case–control study
Gong et al. [24]	2007	China	360	Plasma	Yes	No	Cohort study
Li et al. [25]	2008	China	81	Plasma	Yes	No	Cohort study
Sun et al. [26]	2011	China	62	Plasma	Yes	No	Cohort study
Peng et al. [27]	2009	China	46	Plasma	Yes	No	Case–control study
Zhang et al. [28]	2004	China	20	Plasma	Yes	No	Case–control study
Sun et al. [29]	2009	China	68	Plasma	Yes	No	Cohort study
Sun et al. [30]	2008	China	25	Plasma	Yes	No	Cohort study
Ozyar et al. [31]	2008	Turkey	92	Plasma	Yes	No	Case–control study
Cao et al. [32]	2003	China	76	Plasma	Yes	No	Cohort study
Lo et al. [33]	1999	China	26	Plasma	Yes	No	Case–control study
Fan et al. [34]	2004	China	32	Serum	yes	No	Case–control study
Jiang et al. [35]	2005	China	26	Plasma	Yes	No	Cohort study
Kondo et al. [36]	2004	Japan	45	Serum	Yes	No	Case–control study
Hsiao et al. [37]	2002	China	110	Serum	Yes	No	Cohort study
Chang et al. [38]	2008	China	31	Plasma	Yes	No	Cohort study
Shao et al. [39]	2003	China	90	Plasma	Yes	No	Cohort study
Stoker et al. [40]	2016	Netherlands	147	Plasma	Yes	No	Cohort study
Chan et al. [41]	2014	China	61	Plasma	Yes	No	Cohort study
Shen et al. [42]	2014	China	196	Plasma	Yes	No	Case–control study
Ferrari et al. [43]	2012	Italy	37	Plasma	Yes	No	Cohort study
Li et al. [44]	2017	China	385	Plasma	Yes	No	Case–control study

**Table 2:** Summary measures of test accuracy from the studies included

Study ID	TP	FP	FN	TN	Sensitivity (95% CI)	Specificity (95% CI)	+LR (95% CI)	−LR (95% CI)
Tan et al. (2010)	27	6	9	36	0.75 (0.58–0.88)	0.86 (0.71–0.95)	5.25 (2.44–11.2)	0.29 (0.16–0.52)
Zhu et al. (2006)	45	6	1	54	0.98 (0.88–1.00)	0.9 (0.79–0.96)	9.78 (4.57–20.9)	0.02 (0.00–0.17)
Ma et al. (2011)	62	35	5	172	0.93 (0.83–0.98)	0.8 (0.77–0.88)	5.47 (4.02–7.46)	0.09 (0.04–0.21)
Liao et al. (2008)	7	2	3	10	0.7 (0.35–0.93)	0.83 (0.52–0.98)	4.2 (1.11–15.8)	0.36 (0.14–0.96)
Gong et al. (2007)	70	17	21	252	0.77 (0.67–0.85)	0.94 (0.90–0.96)	12.17 (7.58–19.5)	0.25 (0.17–0.36)
Li et al. (2008)	28	8	2	43	0.93 (0.78–0.99)	0.84 (0.71–0.93)	5.95 (3.13–11.3)	0.08 (0.02–0.30)
Sun et al. (2011)	15	7	2	38	0.88 (0.64–0.99)	0.84 (0.71–0.94)	5.67 (2.81–11.4)	0.14 (0.04–0.52)
Peng et al. (2009)	23	1	2	20	0.92 (0.74–0.99)	0.95 (0.76–1.00)	19.32 (2.84–131.3)	0.08 (0.02–0.32)
Zhang et al. (2004)	8	1	1	10	0.89 (0.52–1.00)	0.91 (0.59–1.00)	9.78 (1.49–64.2)	0.12 (0.02–0.78)
Sun et al. (2009)	25	7	1	35	0.96 (0.80–1.00)	0.83 (0.69–0.93)	5.77 (2.92–11.3)	0.05 (0.01–0.32)
Sun et al. (2008)	14	3	1	7	0.93 (0.68–1.00)	0.7 (0.35–0.93)	3.11 (1.20–8.10)	0.10 (0.01–0.66)
Ozyar et al. (2008)	13	7	1	71	0.93 (0.66–1.00)	0.91 (0.82–0.96)	10.35 (5.03–21.2)	0.08 (0.01–0.52)
Cao et al. (2003)	41	3	11	21	0.79 (0.65–0.89)	0.88 (0.68–0.97)	6.31 (2.17–18.3)	0.24 (0.14–0.42)
Lo et al. (1999)	7	1	3	15	0.7 (0.35–0.93)	0.94 (0.70–1.00)	11.2 (1.61–77.9)	0.32 (0.12–0.83)
Fan et al. (2004)	11	1	2	18	0.85 (0.55–0.98)	0.95 (0.74–1.00)	16.08 (2.35–109.6)	0.16 (0.05–0.58)
Jiang et al. (2005)	19	1	1	5	0.95 (0.75–1.00)	0.83 (0.36–1.00)	5.7 (0.95–34.2)	0.06 (0.01–0.42)
Kondo et al. (2004)	17	2	2	24	0.89 (0.67–0.99)	0.92 (0.75–0.99)	11.63 (3.04–44.4)	0.11 (0.03–0.43)
Hsiao et al. (2002)	10	6	15	79	0.4 (0.21–0.61)	0.93 (0.85–0.97)	5.67 (2.28–14.0)	0.65 (0.47–0.89)
Chang et al. (2008)	8	1	2	20	0.8 (0.44–0.97)	0.95 (0.76–1.00)	16.8 (2.42–116.1)	0.21 (0.06–0.73)
Shao et al. (2003)	29	7	1	53	0.97 (0.83–1.00)	0.88 (0.77–0.95)	8.29 (4.12–16.6)	0.04 (0.01–0.26)
Stoker et al. (2016)	12	19	4	112	0.75 (0.48–0.93)	0.85 (0.78–0.91)	5.17 (3.13–8.55)	0.29 (0.12–0.69)
Chan et al. (2014)	17	1	1	42	0.94 (0.73–1.00)	0.98 (0.88–1.00)	40.61 (5.83–282.3)	0.06 (0.01–0.38)
Shen et al. (2014)	145	1	49	1	0.75 (0.68–0.81)	0.5 (0.01–0.99)	1.49 (0.37–5.99)	0.51 (0.12–2.06)
Ferrari et al. (2012)	5	1	2	29	0.71 (0.29–0.9)	0.97 (0.83–1.00)	21.43 (2.95–155.6)	0.30 (0.09–0.96)
Li et al. (2017)	53	40	19	273	0.74 (0.62–0.83)	0.87 (0.83–0.91)	5.76 (4.18–7.94)	0.30 (0.21–0.45)

**Table 3:** Summary of meta-analysis results

Parameter	Estimate (95% CI)
Sensitivity	0.858 (0.801-0.901)
Specificity	0.890 (0.866-0.909)
Positive Likelihood Ratio	7.782 (6.423-9.429)
Negative Likelihood Ratio	0.159 (0.112-0.226)
Diagnostic Score	3.889 (3.463-4.315)
DOR	48.865(31.903-74.845)

In addition, we used Cochran’s Q test and *I^2^* statistic to assess heterogeneity between studies. Heterogeneity was considered statistically significant when *P*<0.05 or *I^2^* > 50%. According to the results in the Figures, there is a large heterogeneity in the study.

### Publication bias

**Figure 8:**
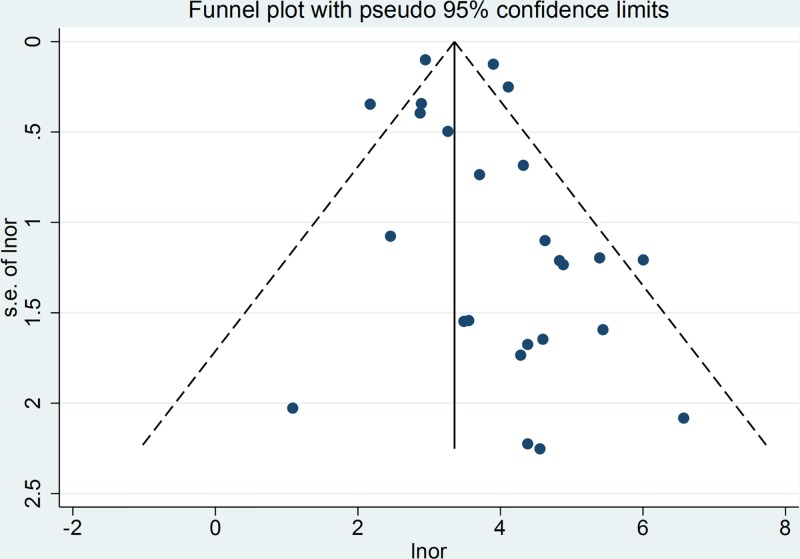
Funnel plot with pseudo 95% confidence limits for EBV DNA assay in the recurrence or/and metastasis of NPC

Funnel plots were performed to evaluate publication bias. Figure 8 shows the asymmetry of the funnel plot of publication bias, indicating the presence of publication bias in the meta-analysis.

### Subgroup analysis

The results of the subgroup analysis are shown in Table 4. The results showed that the source of heterogeneity between studies was independent of the total number of subjects (*n*<30 vs ≥30), study design (case–control vs cohort study), and test specimens (serum vs plasma) (*P*>0.05).

**Table 4:** Subgroup analysis

Grouping situation	Number	Sensitivity	Specificity
Sample			
<30	5	0.83 ± 0.12	0.84 ± 0.09
>30	20	0.83 ± 0.14	0.87 ± 0.10
***P***-value		0.976	0.489
Sample source			
Plasma	22	0.85 ± 0.10	0.86 ± 0.10
Serum	3	0.71 ± 0.27	0.93 ± 0.02
***P***-value		0.481	0.253
Study design			
Cohort study	16	0.84 ± 0.15	0.87 ± 0.07
Case–control study	9	0.82 ± 0.10	0.86 ± 0.14
***P***-value		0.707	0.082

## Discussion

The local recurrence or distant metastasis rate of NPC after 5 years of first-course treatment was 8.2–22.0%, and mainly occurred within 1–3 years after treatment [45,46]. Studies have shown that the survival period for the distant metastasis of NPC is only 12–20 months [47]. During follow-up of patients with NPC, diagnosis of recurrence or metastasis is based on a basic medical history, physical examination, appropriate imaging studies (MRI and/or enhanced CT), and histological examination. PET/CT, as a functional imaging examination, can reflect local tissue metabolism and identify abnormal changes after radiotherapy such as scarring, fibrosis, or tumor recurrence in the diagnosis of the recurrence or metastasis of NPC in patients [48,49]. In addition, PET can be used to determine the correlation between the differentiation degree of NPC through SUV, which could help to confirm the pathological classification of patients who cannot obtain a pathological diagnosis [50]. Nonetheless, local chronic mucosal ulcers, granulomatous tissue, inflammatory changes, and radioactive osteomyelitis formed after radiotherapy inevitably lead to FP results [51]. FN results were also difficult to avoid, as the activity of tumor cells decreases after treatment, and the SUV value decreases accordingly. Furthermore, for lesions less than 1 cm in diameter, the diagnostic sensitivity will be further reduced if 18f-fdg uptake is insufficient [51]. One of the most realistic and important problems is the high cost of PET/CT examination, and the general application during follow-up is inconsistent with the economic development level of China. Hence, it is urgent to find a simple and convenient, economical, highly sensitive, and specific detection method for the early diagnosis of recurrence or metastasis of NPC, and to give more active and appropriate treatment accordingly to guide clinical work.

Mutirangura et al. [52] first detected serum-free EBV DNA by conventional PCR in 1998. Later, Shotelersuk et al. [53] further confirmed the above conclusions by nested-PCR and proposed that free EBV DNA in peripheral blood was obtained from tumor cells. Lo et al. [33] were the first to use RT-PCR technology to study the relationship between the level of plasma EBV DNA and tumor recurrence. The results showed that the level of plasma EBV DNA (median copy number: 32350 copies/ml) in 10 patients with recurrence was significantly higher than that in 15 patients with continuous remission for 2 years (median copy number: zero copies/ml), *P*=0.01. These results indicated that the level of plasma EBV DNA could be used as a reliable indicator for the diagnosis of NPC recurrence or metastasis. Moreover, the value of free EBV DNA in the diagnosis of NPC recurrence or metastasis is also supported by imaging examination. Makitie et al. [54] reported that the detection effects of the copy number of plasma EBV DNA with NPC patients were consistent with the PET/CT examination, both of which were superior to MRI, in the monitoring of NPC local recurrence or distant metastasis.

The present meta-analysis included 25 studies with 2496 patients to evaluate the effectiveness and identify the value of EBV DNA levels as a tool to diagnose the recurrence or metastasis of NPC. Our statistical analysis shows that the pooled sensitivity, specificity, and AUG values achieved 0.858, 0.890, and 0.93, respectively, indicating a very high level of overall accuracy. For post-treatment NPC patients, the specificity value of diagnostic tests during follow-up should be as high as possible to exclude FP diagnosis. According to the results of subgroup analysis, the specific value of serum-derived EBV DNA was the highest, which was 0.933 (95% CI: 0.91–0.95). This means that during follow-up, NPC patients should be selected for serum-derived EBV DNA as much as possible. In statistics, DOR reflects the extent of association between the results of diagnostic tests and diseases. The DOR value was 48.865, indicating a very high discriminant effect in the diagnostic test. Overall, our results suggested that plasma EBV DNA has a high enough accuracy in diagnosing the recurrence or metastasis of NPC.

However, there are several limitations to our study. First, there are publication biases and heterogeneity in the results. After ensuring that the raw data were entered correctly, we conducted a subgroup analysis to explore the sources of heterogeneity and publication bias. Subgroup analysis of possible factors (sample size, sample source, study design) showed that these factors have little impact on the results. This may be related to incomplete information in the selected studies, including disease stage, age distribution, gender distribution etc., which led to the inability of the present study to adequately assess the impact of these variables. In addition, because most of the studies considered recurrence or metastasis as a whole and did not provide specific case data for recurrence or metastasis, the diagnostic efficacy of detecting EBV DNA in the diagnosis of NPC recurrence or metastasis could not be obtained, respectively. Finally, follow-up times are key factors for the accurate diagnosis of the posttreatment clinical remission period. A sufficiently long follow-up time will contribute to distinguishing cases in clinical remission. However, only five of the included studies provided follow-up time data (2–145 months), while most of the studies lacked data on the specific follow-up time, which would affect the accuracy of diagnosis to some extent.

In conclusion, our meta-analysis of currently available data provided reliable evidence that the level of plasma EBV DNA, as a tumor marker for NPC with high sensitivity and good specificity, has a high diagnostic efficacy in the diagnosis of the recurrence or metastasis of NPC. However, it is important to note that the level of plasma EBV DNA cannot replace nasopharyngeal endoscopy and imaging examination as the gold standard for the diagnosis of the recurrence or metastasis of NPC. In the clinic, the frequency of nasopharyngeal endoscopy and imaging examination can be appropriately reduced according to the level of plasma EBV DNA, and the interval time of the above examination can be extended, which is a simple, effective and economical diagnostic method during the follow-up of NPC. These measures are of great significance in improving the therapeutic effect and prognosis of patients with malignant NPC.

## Supporting information

**Supplementary Figure S1:** 

## Abbreviations

CI: confidence interval
CT: computed tomography
DOR: diagnostic odds ratio
EBV: Epstein–Barr virus
FN: false negative
FP: false positive
MRI: magnetic resonance imaging
NPC: nasopharyngeal carcinoma
PET: positron emission tomography
QUADS: quality assessment of diagnostic accuracy studies
SROC: summary receiver operating characteristic
SUV: standard uptake value
TN: true negative
TP: true postive

## References

[1] Marks J.E., Phillips J.L. and Menck H.R. (1998) The National Cancer Data Base report on the relationship of race and national origin to the histology of nasopharyngeal carcinoma. Cancer 83, 582–588 9690553 10.1002/(sici)1097-0142(19980801)83:3<582::aid-cncr29>3.0.co;2-r

[2] Wei W.I. and Sham J.S. (2005) Nasopharyngeal carcinoma. Lancet 365, 2041–2054 10.1016/S0140-6736(05)66698-6 15950718

[3] Parkin D.M., Bray F., Ferlay J. and Pisani P. (2005) Global cancer statistics, 2002. CA Cancer J. Clin. 55, 74–108 10.3322/canjclin.55.2.74 15761078

[4] Jiang G.M., Wang H.S., Du J., Ma W.F., Wang H., Qiu Y. (2017) Bortezomib relieves immune tolerance in nasopharyngeal carcinoma via STAT1 suppression and indoleamine 2,3-dioxygenase downregulation. Cancer Immunol. Res. 5, 42–51 10.1158/2326-6066.CIR-16-0102 27923823

[5] Tan W.L., Tan E.H., Lim D.W., Ng Q.S., Tan D.S., Jain A. (2016) Advances in systemic treatment for nasopharyngeal carcinoma. Chin. Clin. Oncol. 5, 21 10.21037/cco.2016.03.03 27121881

[6] Kam M.K., Teo P.M., Chau R.M., Cheung K.Y., Choi P.H., Kwan W.H. (2004) Treatment of nasopharyngeal carcinoma with intensity-modulated radiotherapy: the Hong Kong experience. Int. J. Radiat. Oncol. Biol. Phys. 60, 1440–1450 10.1016/j.ijrobp.2004.05.022 15590175

[7] Lu T.X. (2004) Advance in diagnosis and management of local recurrent nasopharyngeal carcinoma. Ai Zheng 23, 230–234 14960253

[8] Fountzilas G., Tolis C., Kalogera-Fountzila A., Karanikiotis C., Bai M., Misailidou D. (2005) Induction chemotherapy with cisplatin, epirubicin, and paclitaxel (CEP), followed by concomitant radiotherapy and weekly paclitaxel for the management of locally advanced nasopharyngeal carcinoma. A Hellenic Cooperative Oncology Group phase II study. Strahlenther. Onkol. 181, 223–230 10.1007/s00066-005-1355-1 15827691

[9] Kim Y.I., Han M.H., Cha S.H., Sung M.W., Kim K.H. and Chang K.H. (2003) Nasopharyngeal carcinoma: posttreatment changes of imaging findings. Am. J. Otolaryngol. 24, 224–230 10.1016/S0196-0709(03)00052-8 12884212

[10] Jeyakumar A., Brickman T.M., Jeyakumar A. and Doerr T. (2006) Review of nasopharyngeal carcinoma. Ear Nose Throat J. 85, 168–184, 10.1177/014556130608500313 16615599

[11] Zhang J., Shu C., Song Y., Li Q., Huang J. and Ma X. (2016) Epstein-Barr virus DNA level as a novel prognostic factor in nasopharyngeal carcinoma: a meta-analysis. Medicine (Baltimore) 95, e5130 10.1097/MD.0000000000005130 27749596 PMC5059099

[12] Sham J.S., Wei W.I., Kwan W.H., Chan C.W., Kwong W.K. and Choy D. (1990) Nasopharyngeal carcinoma. Pattern of tumor regression after radiotherapy. Cancer 65, 216–220 2295044 10.1002/1097-0142(19900115)65:2<216::aid-cncr2820650206>3.0.co;2-z

[13] Chua M., Wee J., Hui E.P. and Chan A. (2016) Nasopharyngeal carcinoma. Lancet 387, 1012–1024 10.1016/S0140-6736(15)00055-0 26321262

[14] Lo Y.M., Chan L.Y., Chan A.T., Leung S.F., Lo K.W., Zhang J. (1999) Quantitative and temporal correlation between circulating cell-free Epstein-Barr virus DNA and tumor recurrence in nasopharyngeal carcinoma. Cancer Res. 59, 5452–5455 10554016

[15] Lo Y.M., Leung S.F., Chan L.Y., Chan A.T., Lo K.W., Johnson P.J. (2000) Kinetics of plasma Epstein-Barr virus DNA during radiation therapy for nasopharyngeal carcinoma. Cancer Res. 60, 2351–2355 10811107

[16] Lin J.C., Wang W.Y., Chen K.Y., Wei Y.H., Liang W.M., Jan J.S. (2004) Quantification of plasma Epstein-Barr virus DNA in patients with advanced nasopharyngeal carcinoma. N. Engl. J. Med. 350, 2461–2470 10.1056/NEJMoa032260 15190138

[17] Chan A.T., Lo Y.M., Zee B., Chan L.Y., Ma B.B., Leung S.F. (2002) Plasma Epstein-Barr virus DNA and residual disease after radiotherapy for undifferentiated nasopharyngeal carcinoma. J. Natl. Cancer Inst. 94, 1614–1619 10.1093/jnci/94.21.1614 12419787

[18] Whiting P., Rutjes A.W., Reitsma J.B., Bossuyt P.M. and Kleijnen J. (2003) The development of QUADAS: a tool for the quality assessment of studies of diagnostic accuracy included in systematic reviews. BMC Med. Res. Methodol. 3, 25 10.1186/1471-2288-3-25 14606960 PMC305345

[19] Higgins J.P., Thompson S.G., Deeks J.J. and Altman D.G. (2003) Measuring inconsistency in meta-analyses. BMJ 327, 557–560 10.1136/bmj.327.7414.557 12958120 PMC192859

[20] Sun J., Wang H., Xiao F. and LIU Y. (2010) The clinical value of plasma EBV DNA, serum cyfra21-1 and vca-iga in monitoring the recurrence and metastasis of nasopharyngeal carcinoma. Chin. Otolaryngol. Head Neck Surg. 2010, 125–127

[21] Tan J., Liao Y., Cao X., Sun S. and Wu L. (2010) The value of quantitative determination of EB virus in plasma in monitoring recurrence and metastasis of nasopharyngeal carcinoma after radiotherapy. J. Hubei Univ. Nationalities (Medical) 27, 20–21

[22] Peng J., Liao H., Lin Z. and Xiao M. (2009) Significance of detection of plasma EB virus DNA level in patients with recurrent and metastatic nasopharyngeal carcinoma. Chin. J. Gen. Pract. 2009, 570–571

[23] Zhu X., Du B., Huang X., Lin S. and Lan Y. (2006) Quantitative detection of EB virus DNA in plasma for the diagnosis and prognosis of nasopharyngeal carcinoma. Chin. J. Otolaryngol. 2006, 73–75

[24] Sun W. and Jin Y. (2008) Detection and clinical application of plasma EBV-DNA, serum CYFRA21-1 and TSGF in patients with nasopharyngeal carcinoma. J. Modern Oncol. 2008, 537–540

[25] Ma D., Li Y. and He X. (2011) Clinical value of quantitative detection of EB virus DNA in plasma in the diagnosis and treatment of nasopharyngeal carcinoma. Lab. Med. Clinic 8, 1978–1980

[26] Liao Y. and Lu Z. (2008) Quantitative determination of EB virus DNA in plasma in patients with nasopharyngeal carcinoma. Chin. Otolaryngol. Head Neck Surg. 2008, 206

[27] Gong X., Li J., Yuan X., Liao Z., Ao F., Zou X. (2007) The clinical significance of plasma EBV DNA level in monitoring the recurrence and metastasis of nasopharyngeal carcinoma after radiotherapy. Pract. J. Cancer 2007, 150–153

[28] Li D., Peng K., Jiang H., Liu J. and Shen X. (2008) The value of plasma EBV DNA quantitative analysis in monitoring the recurrence and metastasis of nasopharyngeal carcinoma. J. Pract. Oncol. 2008, 12–16

[29] Sun J. and Zheng A. (2008) The clinical significance of plasma EBV DNA level and VCA-IgA in patients with nasopharyngeal carcinoma. J. Modern Oncol. 16, 2086–2087

[30] Zhang L., Li S., Zhao C., Peng J., Huang P. and Li W. (2004) Relationship between plasma EB virus DNA level and tumor recurrence in patients with nasopharyngeal carcinoma. J. Clin. Oncol. 2004, 122–125

[31] Ozyar E., Gultekin M., Alp A., Hascelik G., Ugur O. and Atahan I.L. (2007) Use of plasma Epstein-Barr virus DNA monitoring as a tumor marker in follow-up of patients with nasopharyngeal carcinoma: preliminary results and report of two cases. Int. J. Biol. Markers 22, 194–199 10.1177/172460080702200305 17922462

[32] Cao S.M., Min H.Q., Gao J.S., Hong M.H., Xiao X.B., Zhang C.Q. (2003) Significance of cell-free Epstein-Barr virus DNA in monitoring prognosis of nasopharyngeal carcinoma. Ai Zheng 22, 302–306 12654192

[33] Lo Y.M., Chan L.Y., Lo K.W., Leung S.F., Zhang J., Chan A.T. (1999) Quantitative analysis of cell-free Epstein-Barr virus DNA in plasma of patients with nasopharyngeal carcinoma. Cancer Res. 59, 1188–1191 10096545

[34] Fan H., Nicholls J., Chua D., Chan K.H., Sham J., Lee S. (2004) Laboratory markers of tumor burden in nasopharyngeal carcinoma: a comparison of viral load and serologic tests for Epstein-Barr virus. Int. J. Cancer 112, 1036–1041 10.1002/ijc.20520 15386346

[35] Jiang W. and Liao Y. (2005) Dynamic study on the relationship between EB virus DNA and nasopharyngeal carcinoma. J. Clin. Otorhinolaryngol. 2005, 920–922 16398045

[36] Kondo S., Horikawa T., Takeshita H., Kanegane C., Kasahara Y., Sheen T.S. (2004) Diagnostic value of serum EBV-DNA quantification and antibody to viral capsid antigen in nasopharyngeal carcinoma patients. Cancer Sci. 95, 508–513 10.1111/j.1349-7006.2004.tb03241.x 15182432 PMC11159009

[37] Hsiao J.R., Jin Y.T. and Tsai S.T. (2002) Detection of cell free Epstein-Barr virus DNA in sera from patients with nasopharyngeal carcinoma. Cancer 94, 723–729 11857305 10.1002/cncr.10251

[38] Shao J.Y., Li Y.H., Gao H.Y., Wu Q.L., Cui N.J., Zhang L. (2004) Comparison of plasma Epstein-Barr virus (EBV) DNA levels and serum EBV immunoglobulin A/virus capsid antigen antibody titers in patients with nasopharyngeal carcinoma. Cancer 100, 1162–1170 15022282 10.1002/cncr.20099

[39] Chang K.P., Hsu C.L., Chang Y.L., Tsang N.M., Chen C.K., Lee T.J. (2008) Complementary serum test of antibodies to Epstein-Barr virus nuclear antigen-1 and early antigen: a possible alternative for primary screening of nasopharyngeal carcinoma. Oral Oncol. 44, 784–792 10.1016/j.oraloncology.2007.10.003 18206420

[40] Chan J.Y. and Wong S.T. (2014) The role of plasma Epstein-Barr virus DNA in the management of recurrent nasopharyngeal carcinoma. Laryngoscope 124, 126–130 10.1002/lary.24193 23686740

[41] Ferrari D., Codeca C., Bertuzzi C., Broggio F., Crepaldi F., Luciani A. (2012) Role of plasma EBV DNA levels in predicting recurrence of nasopharyngeal carcinoma in a western population. BMC Cancer 12, 208 10.1186/1471-2407-12-208 22646734 PMC3443044

[42] Stoker S.D., Wildeman M.A., Novalic Z., Fles R., van der Noort V., de Bree R. (2016) Can Epstein-Barr virus DNA load in nasopharyngeal brushings or whole blood predict recurrent nasopharyngeal carcinoma in a non-endemic region? A prospective nationwide study of the Dutch Head and Neck Oncology Cooperative Group Eur. Arch. Otorhinolaryngol. 273, 1557–1567 10.1007/s00405-015-3620-y 25929413

[43] Shen T., Tang L.Q., Luo D.H., Chen Q.Y., Li P.J., Mai D.M. (2015) Different prognostic values of plasma Epstein-Barr virus DNA and maximal standardized uptake value of 18F-FDG PET/CT for nasopharyngeal carcinoma patients with recurrence. PLoS ONE 10, e122756 10.1371/journal.pone.0122756 PMC4390333 25853677

[44] Li W.F., Zhang Y., Huang X.B., Du X.J., Tang L.L., Chen L. (2017) Prognostic value of plasma Epstein-Barr virus DNA level during posttreatment follow-up in the patients with nasopharyngeal carcinoma having undergone intensity-modulated radiotherapy. Chin. J. Cancer 36, 87 10.1186/s40880-017-0256-x 29116021 PMC5678814

[45] Lee A.W., Sze W.M., Au J.S., Leung S.F., Leung T.W., Chua D.T. (2005) Treatment results for nasopharyngeal carcinoma in the modern era: the Hong Kong experience. Int. J. Radiat. Oncol. Biol. Phys. 61, 1107–1116 10.1016/j.ijrobp.2004.07.702 15752890

[46] Xiao W.W., Han F., Lu T.X., Chen C.Y., Huang Y. and Zhao C. (2009) Treatment outcomes after radiotherapy alone for patients with early-stage nasopharyngeal carcinoma. Int. J. Radiat. Oncol. Biol. Phys. 74, 1070–1076 10.1016/j.ijrobp.2008.09.008 19231110

[47] Chan A.T. (2010) Nasopharyngeal carcinoma. Ann. Oncol. 21, i308–i312 10.1093/annonc/mdq277 20943634

[48] Ahn P.H. and Garg M.K. (2008) Positron emission tomography/computed tomography for target delineation in head and neck cancers. Semin. Nucl. Med. 38, 141–148 10.1053/j.semnuclmed.2007.11.002 18243850

[49] Connell C.A., Corry J., Milner A.D., Hogg A., Hicks R.J., Rischin D. (2007) Clinical impact of, and prognostic stratification by, F-18 FDG PET/CT in head and neck mucosal squamous cell carcinoma. Head Neck 29, 986–995 10.1002/hed.20629 17563906

[50] Liu F., Xi X.P., Wang H., Han Y.Q., Xiao F., Hu Y. (2017) PET/CT-guided dose-painting versus CT-based intensity modulated radiation therapy in locoregional advanced nasopharyngeal carcinoma. Radiat. Oncol. 12, 15 10.1186/s13014-016-0739-y 28587681 PMC5461636

[51] Liu S.H., Chang J.T., Ng S.H., Chan S.C. and Yen T.C. (2004) False positive fluorine-18 fluorodeoxy-D-glucose positron emission tomography finding caused by osteoradionecrosis in a nasopharyngeal carcinoma patient. Br. J. Radiol. 77, 257–260 10.1259/bjr/69516821 15020372

[52] Mutirangura A., Pornthanakasem W., Theamboonlers A., Sriuranpong V., Lertsanguansinchi P., Yenrudi S. (1998) Epstein-Barr viral DNA in serum of patients with nasopharyngeal carcinoma. Clin. Cancer Res. 4, 665–669 9533535

[53] Shotelersuk K., Khorprasert C., Sakdikul S., Pornthanakasem W., Voravud N. and Mutirangura A. (2000) Epstein-Barr virus DNA in serum/plasma as a tumor marker for nasopharyngeal cancer. Clin. Cancer Res. 6, 1046–1051 10741733

[54] Makitie A.A., Reis P.P., Irish J., Zhang T., Chin S.F., Chen X. (2004) Correlation of Epstein-Barr virus DNA in cell-free plasma, functional imaging and clinical course in locally advanced nasopharyngeal cancer: a pilot study. Head Neck 26, 815–822 10.1002/hed.20028 15350028

