# Supplementary figures and images for: Clinical value of a plasma Epstein–Barr virus DNA assay in the diagnosis of recurrent or metastatic nasopharyngeal carcinoma: a meta-analysis
Source: Biosci Rep. 2019 Sep 20;39(9):BSR20190691. doi: 10.1042/BSR20190691 (PMC6753325; doi:10.1042/BSR20190691)

# Fagan's Nomogram

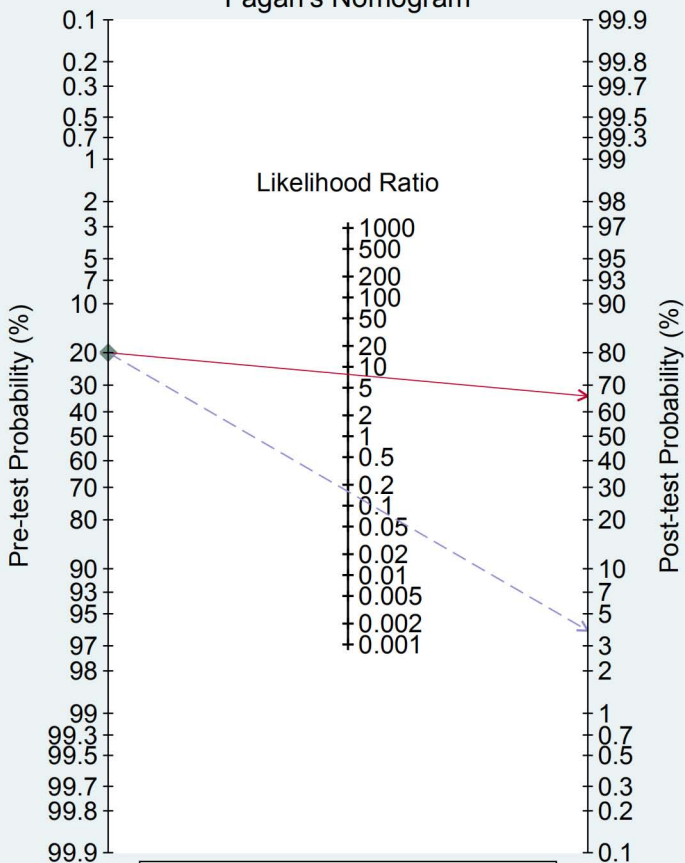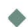

Prior Prob (%) = 20

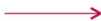

LR\_Positive = 8  
Post\_Prob\_Pos (%) = 66

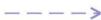

LR\_Negative = 0.16  
Post\_Prob\_Neg (%) = 4

Supplement: Supplementary file 1 [file bsr20190691_Supp1.pdf]
